# Depression Treatment: Is There a Role for Botulinum Toxin Type A?

**DOI:** 10.3390/microorganisms12122615

**Published:** 2024-12-17

**Authors:** Carmen Rodríguez-Cerdeira, Westley Eckhardt

**Affiliations:** 1 Department of University Program for Seniors, University of Vigo, E.E. Industrial Rúa Torrecedeira 86, Vigo Campus, 36201 Vigo, Spain; m72433337@gmail.com; 2 Fundación Vithas, Grupo Hospitalario Vithas, 28043 Madrid, Spain; 3 Dermatology Department, Grupo Hospitalario (CMQ Concheiro), Manuel Olivié 11, 36203 Vigo, Spain

**Keywords:** antidepressant, botulinum toxin type A, depression, systematic review

## Abstract

This study aimed to determine whether botulinum toxin type A injected into the muscles of the upper third of the face has antidepressant effects in patients diagnosed with depression. Studies seeking a relationship between botulinum toxin type A and its antidepressant effects were considered in this review. All studies concluded that the facial expression muscles present positive feedback to the brain and enhance mood states. Botulinum toxin when applied to the corrugator and procerus muscles has an antidepressant effect.

## 1. Introduction

Depression affects 5% of the global population [1], and its rates of disability and morbidity have continued to increase, especially during the coronavirus disease (COVID) pandemic. Depressive episodes usually occur in patients with MDD and bipolar disorder (BD). The treatment of bipolar depression is challenging. Depressive episodes are frequently undertreated or undiagnosed, and those diagnosed in approximately half of the cases remain untreated [2].

Objective quantification of patient symptoms to monitor treatment response remains challenging. The Patient Health Questionnaire–9 (PHQ-9) is an established rating scale for assessing symptom severity and screening for depression in clinical practice [3]. Other scales, such as the Hamilton Depression Rating Scale (HAM-D), are also commonly utilized. Significant symptom improvement is defined as an improvement of at least 50% from baseline scores, while remission is characterized by subthreshold scores [4].

Pharmacotherapy is the favored evidence-based treatment for moderate-to-severe depression, supported by meta-analyses of randomized controlled trials. Selective serotonin reuptake inhibitors (SSRIs) and serotonin–norepinephrine reuptake inhibitors (SNRIs) are commonly prescribed. However, half of the patients report bothersome side effects from these medications. Other medications outside these two categories of antidepressants demonstrate efficacy but can also lead to adverse effects [5,6].

Another evidence-based treatment for MDD is psychotherapy, with interpersonal therapy and cognitive behavioral therapy (CBT) proving particularly effective for mild to moderate depressive episodes. This treatment involves weekly or twice-weekly sessions lasting 8 to 16 weeks, which can pose access barriers to effective psychotherapy. Most patients receiving pharmacotherapy or psychotherapy experience symptom improvement or remission within 2–4 months; however, 30% of patients do not respond to these treatments, classifying them as having treatment-resistant depression. Patients with treatment-resistant depression have higher rates of hospitalization and suicide attempts [7].

Somatic interventions, such as electroconvulsive therapy, repetitive transcranial magnetic therapy, vagus nerve stimulation, and deep brain stimulation, can be alternative treatments for treatment-resistant depression. Electroconvulsive therapy has been studied for decades and has shown rapid antidepressant effects, though retrograde amnesia is a common side effect. Repetitive transcranial magnetic therapy is effective for moderate depression; however, daily sessions are an obstacle. Vagus nerve stimulation yields positive results but is invasive and costly. Deep brain stimulation shows promise for treatment-resistant depression but requires surgically implanted electrodes, which can lead to adverse neurological effects [8,9].

Recent developments in treating treatment-resistant depression, including intravenous ketamine injections, have shown success in improving symptoms. Additionally, psilocybin promises rapid improvement for depressive symptom; however, further studies are warranted [7,9].

Botulinum toxin (BoNT), a neurotoxin produced by the bacterium *Clostridium botulinum*, is capable of inducing progressive muscular paralysis. It has been traditionally used to treat various diseases such as spasticity, pain, and bruxism; however, recent studies have proposed the use of BoNT in patients with depressive disorders [10]. For example, a voluntary contraction of the facial muscles during a smile or frown can induce feelings of happiness or sadness, respectively [11]. The theory of its effectiveness began with Charles Darwin’s theory of facial expressions of emotions in animals and humans [12], which Carl Georg Lange further supported through his facial feedback theory of emotion [13].

Paul Ekman et al. [14] clarified whether altering facial expressions affected the autonomic nervous system. Their work showed that heart rate, finger temperature, and skin resistance increase with anger, fear, and sadness. Other research groups tested how the muscles of facial expression affected emotional states [14,15].

Facial expressions of negative emotions, such as fear, sadness, and anger, associated with the contraction of the corrugator muscles, have been implicated in depression in several studies [16,17,18]. BoNT injection targets these muscles by reversibly blocking acetylcholine release from neuronal axons at synapses, thereby inhibiting neuromuscular transmission [19]. While the idea that treating wrinkles with BoNT may improve the mood of patients with depression seems farfetched, the rationale for this treatment is grounded in the long-established facial feedback theory. It has been suggested that when people make facial expressions, feedback from the muscles of the face can modulate the subjective experience of emotions [20,21].

**Figure 1:**
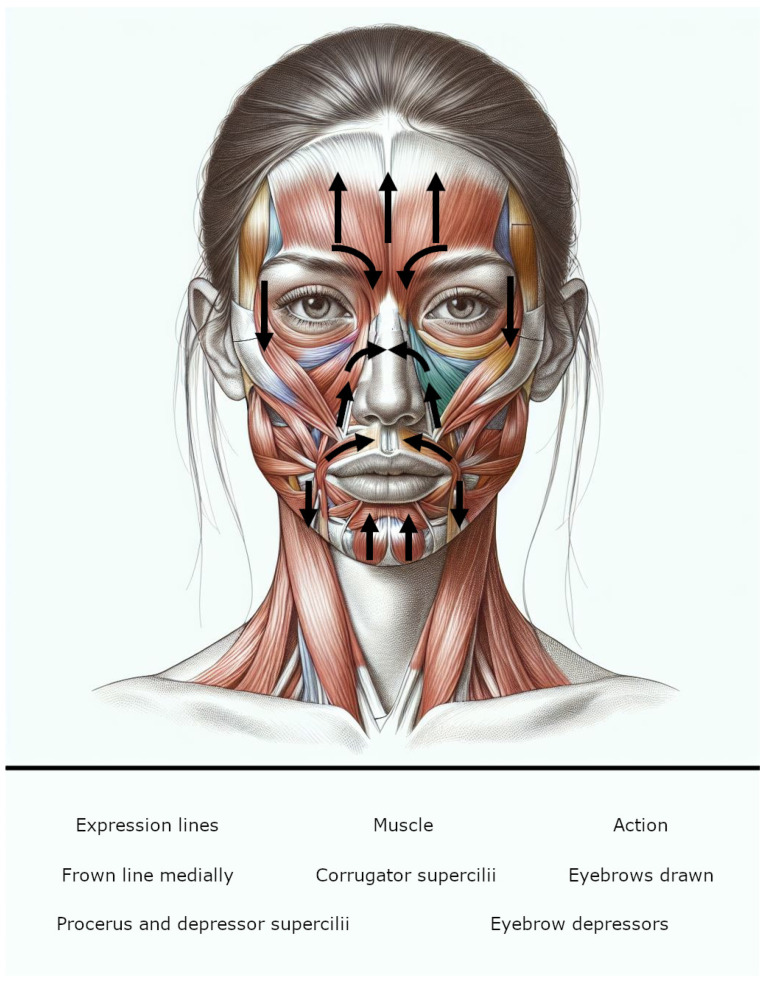
A positive feedback loop exists between facial expression muscles and the brain. When specific muscles contract to express sadness, the brain interprets this information as true sadness and reinforces this feeling in the person.

BoNT is often used to treat muscle spasms, multiple sclerosis, and incontinence. Notably, onabotulinum A (OnNT/A) injections have also been shown to be effective against chronic migraines and major depressive disorders [22]. The proposed mechanism for its antidepressant effects involves blocking feedback from facial expression, particularly negative emotions that cause the corrugator and procerus muscles to contract in the forehead, resulting in frowning (Figure 1) [23].

## 2. Materials and Methods

An advanced search was performed in English and Spanish using the following databases: Medical Literature Analysis and Retrieval System Online (MEDLINE/PubMed), Scientific Electronic Library Online (SciELO), Google Scholar, and SCOPUS. The search targeted case reports, observational studies, and clinical trials from March 1993 to May 2024. In addition, we included references from previous years that were considered to be of practical relevance.

The following search terms were used to identify potential articles: [“botulinum toxin type A” AND “BoNT/A” OR “botox” OR “onabotulinum A”] and [“antidepressant effect” AND “depression” OR “mood disorder” OR “emotional regulation” OR “affect”].

**Figure 2:**
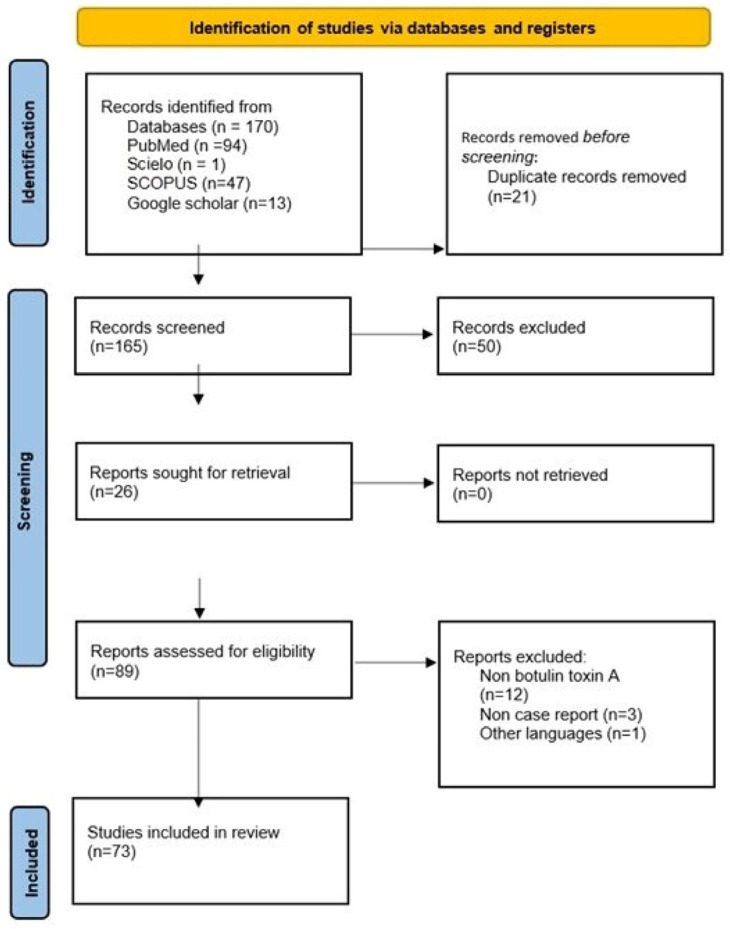
PRISMA 2020 flow diagram for new systematic reviews, which include searches of databases and registers.

A total of 155 articles were found, and the review was carried out based on the Preferred Reporting Items for Systematic Reviews and Meta-Analyses (PRISMA) (see Figure 2) [24]. To refine the selection, search criteria included case reports, case series, observational studies, and clinical trials. After screening the titles and analyzing the full texts, the most relevant articles for review that met the inclusion and exclusion criteria were selected. Finally, 55 original articles were selected for analysis. However, this review was limited to articles that offered detailed descriptions of fungal melanonychia, diagnostic methods, and treatment approaches.

Although authors in various published works use other abbreviations, to unify the criteria, we will use BoNT/A to refer to botulinum toxin type A and OnNT/A to refer to onabotulinum A.

## 3. Results and Discussion

Many randomized controlled trials have shown strong evidence that BoNT/A is effective for treatment-resistant depression. BoNT/A treatment for MDD and treatment-resistant depression is generally safe, tolerable, and long-lasting. As it requires only a few administrations annually, its cost remains comparable to that of branded medications and is lower than therapies necessitating frequent clinical visits. Therefore, BoNT/A is an acceptable treatment option for patients with treatment-resistant depression [25].

Strack et al. [26] studied whether facial feedback influenced emotion perception. The participants were asked to hold a pen with their lips or teeth only. The rationale for holding a pen in their lips was that this would “support contraction of the zygomaticus major or risorius muscles that are used during smiling”, while holding a pen in their teeth would “primarily contract” the same muscles that are involved in smiling. This is consistent with the idea that activating muscles associated with smiling could enhance positive emotions via feedback mechanisms [27]. The authors explored the facial feedback effect in three experiments. They found that lowering someone’s eyebrows led to a more negative mood, while raising them induced a feeling of surprise. Finally, when participants were instructed to wrinkle their noses, odors were evaluated as more pleasant. These findings reinforce the facial feedback theory, particularly in the context of facial muscular paralysis from cosmetic treatments using BoNT/A. This research supports the previously suggested idea that such treatments could reduce depression; however, other possible psychological effects of such treatments have been considered [28].

Larsen et al. [29] extended this approach to negative expressions. To lead the participants to furrow their eyebrows without asking them to do so, two golf tees were placed on the participants’ eyebrows just above the inner corner of each eye. They were asked to touch the tips of golf tees or to hold them apart as still as possible as part of a divided attention experiment. Touching the tips involves contraction of the corrugator muscles, which causes furrowing between the eyebrows, resulting in a sad expression. Consequently, frowning causes a decrease in mood disorders [30].

Given that certain facial muscles can alter mood perception, just as frowning can decrease mood, the next pertinent question is whether paralyzing facial muscles can prevent mood disorders [31].

Wollmer et al. [32] introduced a new treatment approach to relieve depression based on emotional proprioception. Despite various psychiatric and pharmaceutical treatment options, approximately one-third of individuals with depression experience incomplete symptom relief, highlighting the need for alternative therapeutic approaches. The glabellar area of the face, with its corrugator and procerus muscles, is critical in expressing emotions such as anger, fear, and sadness, which are often seen in depression. The facial wrinkles in the human brow that resemble the Greek letter omega are associated with stress, mental disturbances, and depression. Both Charles Darwin and William James proposed the facial feedback hypothesis that facial expressions of emotions provoke proprioceptive feedback signals that maintain the expressed emotions. This emotional feedback loop may be interrupted by relaxing the glabellar muscles [33].

To date, four small trials with 28–74 participants, mostly women, have been reported. Several meta-analyses, albeit in small numbers, have also been performed. Findings indicate that BoNT injections in the glabellar region exhibit anti-depressant effects, with successful outcomes observed in most women with unipolar depression and some men with bipolar depression.

Further studies on associated conditions treated with BoNT, such as chronic migraines, torticollis, blepharospasm, and hyperhidrosis, are likely to have comorbid depression. Mouse models of depression respond to single doses of BoNTs.

BoNT serves as a ready-to-use tool for assessing and treating depression. However, it remains impossible to predict who will benefit from this treatment as prospective patients do not necessarily need to have visible frown lines. Its efficacy in relieving depression symptoms is grounded on the rationale that at higher doses, its muscle relaxing effect, leads to positive facial expressions, reinforcing the placebo effect associated with the treatment [34].

Li et al. [35] highlighted several mechanisms through which BoNTs/A induce anti-depressant effects. Their research supports the interruption of emotional proprioception and the facial feedback hypothesis, suggesting the involvement of mechanical receptors on the face or molecular mechanisms. Contributing factors may include placebo effects, enhanced body image, increased self-esteem, and improved social interaction. They also suggested targeting injection sites outside the face [35].

Various mechanisms have been implicated in the action of BONT/As. BDNF plays a functional role in neuronal differentiation and survival, neurogenesis, synaptic plasticity, connectivity, and the maintenance of morphology, learning, and memory [36]. Furthermore, BDNF levels in several brain regions are markedly reduced in depression-like animals and patients with depression. Several commonly used antidepressants, such as SSRIs [37], upregulate BDNF expression in the hippocampus and prefrontal cortex to exert antidepressant effects [38].

Other studies have shown that facial injection of BoNT/A can upregulate the expression of BDNF in the hippocampus of mice at the mRNA and protein levels.

Additionally, BoNT/A injection activates the ERK-CREB signaling pathway downstream of BDNF in the hippocampus of mice previously subjected to stress [39].

Castren et al. [40] proposed the monoamine theory, where patients with emotional disorders are associated with reduced availability of 5-hydroxytryptamine (5-HT) and norepinephrine (NE) in their brain [40].

It is proven that the use of facial injections of BoNT/A significantly increased 5-HT levels in the hippocampus, hypothalamus, and prefrontal cortices of chronically stressed mice [41].

Extensive research has been conducted on the insular, prefrontal, and anterior cingulate cortices.

The amygdala and hippocampus play key roles in processing sensations and emotions [42].

Thus, BoNT/A may play integrative and transdiagnostic roles in mental disorders, showing effectiveness in cases characterized by excessive negativity, including borderline personality disorder (BDP) and social anxiety disorder (SAD). The BoNT/A treatment is a bottom-up treatment unlike traditional top-down depression treatments. BoNT integrates pharmacotherapy, relaxation exercises, behavioral therapy, and social therapy. Unfortunately, research has largely stalled at phase II due to delays caused by the COVID-19 pandemic [43].

Randomized controlled trials (RCTs) and meta-analyses have shown that glabellar BoNT/A injection moderates depressive symptoms. As phase III studies are still pending, BoNTs have not been registered as a formal treatment for depression. However, as a registered treatment for frown lines, BoNT/A can be used to manage depression [44].

Recently, an investigator-borne initiative (https://www.healisthera.com accessed on 5 July 2024) was founded to rapidly develop BoNT therapy for depression towards registration [45].

Coles et al. [46], through the presence of emotional stimuli, verified that the effects of facial feedback on emotional experience were greater in the absence of emotionally evocative stimuli (e.g., cartoons). The available evidence supports the central claim of the facial feedback hypothesis that facial feedback influences emotional experiences. In their meta-analysis, they provided considerable evidence and concluded that smiles, frowns, grimaces, and other facial movements could affect patients’ emotions in various situations.

Finzi et al. [47] reported that studies based on electromyographic measures of corrugator muscle activity demonstrated an increase in depression. This prompted researchers to explore whether electromyography patterns of facial muscle activity could predict outcomes in patients with depression, and evidence suggests that they can. This process is referred to as “emotional proprioception”, in which nerve fibers from the face relay information to the brain. The fundamental connection is between the corrugator muscles and the amygdala, which directly influences negative emotions. Amygdalar responses are decreased after injecting BoNT/A into the procerus and corrugator muscles, highlighting an emotional proprioceptive mechanism of BoNT/A’s antidepressant activity.

To date, 10 trials conducted across five countries demonstrated significant improvements in depression and anxiety symptoms with BoNT/A treatment. Five meta-analyses of clinical trials confirmed the strong antidepressant effect of BoNT/A compared to placebo. The FDA adverse effect reporting system used to review whether 40,000 BoNT/A treatments reduced the incidence of depression and/or anxiety showed significantly lower incidences [48]. As one-third of the patients with depression fail to respond to oral antidepressants and psychotherapy, new treatments are urgently needed. BoNT/A is a potential treatment option with several well-established advantages including minimal equipment requirements, ease of delivery and administration, widespread experience among medical professionals with injections, and prolonged effectiveness (lasting 3 months or more), thus reducing the frequency of visits and enhancing adherence [48].

Thirty years of experience with BoNT/A therapy and cosmetic use in millions of patients have demonstrated its safety and tolerability in glabellar frown muscles, with only very mild side effects. The burden of these side effects is significantly lower than that of other treatments for depression and anxiety. BoNT/A shows potential for treating many psychiatric disorders beyond MDD, bipolar depression [49], social anxiety, panic, post-traumatic stress, borderline personality, and obsessive-compulsive disorders, all of which involve amygdalar dysregulation. This suggests a new paradigm for psychiatric treatment [50].

As reported by Finzi et al. [51], BoNT/A appears to be a good treatment option for patients with treatment-resistant depression or difficult episodes of bipolar depression, demonstrating its safety and tolerability. Unfortunately, the mechanisms underlying its efficacy remain unclear, posing challenges for justifying further research. Many treatments for depression, including SSRIs, remain poorly understood, particularly regarding their delayed antidepressant actions, which lack a robust biological explanation. The medical understanding of antidepressant mechanisms remains incomplete. Further research is needed to investigate whether BoNT/A is suitable for bipolar depression treatment, despite uncertainties regarding its biological basis. Nevertheless, it offers a safe and tolerable alternative that merits further research as a viable treatment modality.

Regarding sex, they found that patients had a remission rate of >50% following OnNT/A treatment. Females showed a higher rate of remission than males. However, this study lasted only 6 weeks [51].

In the search conducted by Parsaik et al. [52], only randomized controlled trials were included in the meta-analysis evaluating the effects of BoNT/A on depression. The overall odds ratio for the combined effect of both the drug and placebo, with a 95% confidence interval, was estimated using a variance components model. A weighted sum of the differences, in primary depression scores and heterogeneity, was estimated using the χ2 statistic and the Cochran Q test. The remission and response rates were 8.3 and 4.6 times greater, respectively, among those treated with BoNT/A compared to placebo, and no heterogeneity between the studies. A few treated patients experienced minor side effects, similar to those in the groups receiving placebo and BoNT/A.

The research conducted by Affatato et al. [53] is the first to analyze the mean effect of OnNT/A injections in patients with comorbid depression and migraine. This meta-analysis evaluated the treatment effects on patients with both disorders and compared them with those who have only one of the two conditions. The studies selected followed the same protocol for OnNT/A injections but differed regarding the scales assessing the impact and severity of migraine and depression and the treatment duration (minimum 3 months, maximum 27 months). Due to these differences, not all studies could be pooled for statistical analysis. The studies were divided into subgroups according to the disease scale for each disorder and treatment duration, with three subgroups for each disorder. The database search yielded 370 studies, from which eight studies were included in the meta-analysis [54].

When OnNT/A is administered to patients with comorbid migraine and MDD, it results in significant symptom reduction for both illnesses. Comparative analysis between monomorbid and comorbid individuals show a similar strong effect and specifically beneficial effects for some migraine features.

Crowley et al. [55] conducted a systematic review and meta-analysis assessing the effectiveness of OnNT/A injections in relieving depression. There are various treatments for MDD, including medications such as selective SSRIs and/or psychotherapy. Despite advances in treatment, treatment resistance remains an issue. Glabellar OnNT/A may be a safe and effective treatment option for MDD.

Hexsel et al. [56] reported its safety history and beneficial effects on depression and attempted to summarize the evidence for glabellar BoNT/A injections as an additional treatment for depression [56].

This meta-analysis and systematic review aimed to illustrate the mean differences in primary depression scores between patients receiving glabellar BoNT/A injections and their placebo-treated counterparts. Only RCTs and non-randomized cohort studies assessing the efficacy of glabellar BoNT/A injections for treating MDD were considered. Only articles employing standard depression scoring scales, such as the Hamilton Depression Rating Scale (HAMD), Beck Depression Inventory (BDI), and Montgomery–Asberg Depression Rating Scale (MADRS) were considered. All studies included were published in English only [57].

All studies used the aforementioned depression rating scales, with the exception of one that used the Hospital Anxiety and Depression Scale. All study participants, except one, received a single BoNT/A or placebo treatment at the outset of the study. Doses were adjusted for men because of their higher muscle mass: 29U for women and 39U for men [58].

The outcomes reported in nine studies in the systematic review showed improvements in mean depression scores from baseline to the first endpoint. All RCTs reported mean depression score improvements compared with the placebo group at 6 weeks post-treatment. The differences were significant in all studies except for one. All nine studies showed a reduction in MDD. The BoNT/A treatments in all nine studies were associated with a 1.61 standard deviation reduction in depression scores. These findings indicate that glabellar OnNT/A injections are an effective treatment for major depressive disorders [59].

The mechanisms underlying the mood-enhancing effects of BoNT/A remain unclear. This may be due to facial feedback or the inhibition of negative proprioception signals from the procerus and corrugator muscles. Other effects may include better cosmesis, positive social feedback, and biochemical effects of OnNT/A on the central nervous system. There were few side effects and no long-term adverse effects related to OnNT/A, except for neuromuscular disorders. The only salient limitation of studies with control groups was that patients were more likely to identify whether they received a placebo when facial paralysis was absent [60].

These studies indicate that glabellar OnNT/A injections are a safe and effective treatment option for MDD. Such treatments may be particularly useful for those who are unable to tolerate antidepressants or those resistant to current depression treatments.

New applications of BoNT/A therapy continue to be explored and developed, thus promising further innovations. Dressler et al. [61] noted that BoNT/A therapy is highly effective and safe across various medical fields and aesthetic applications. Further improvements in treatment algorithms and expansion of indications, potentially for conditions such as depression and arthritis, is likely. However, its market penetration is insufficient, and a large number of patients who could benefit from BoNT/A have not yet been identified.

Several factors contributed to this failure, including a lack of awareness, education, training, and therapy costs. The current business model, based on high prices for BoNT/A treatment, limits access for those who cannot afford the treatment. A biosimilar registration approach could help reduce the cost of registered botulinum toxin formulae or therapies, especially in countries where business models are not dominated by the pharmaceutical industry or private medicine.

In a review and meta-analysis [62], evidence supporting BoNT/A as a treatment for MDD has been established. BoNT/A has been used in cosmetic treatments of facial wrinkles in the glabellar region for more than 30 years. Studies have shown that BoNT/A negatively affects emotional expression. The same negative effect can be used to reduce negative emotions, such as in major depressive disorders [63].

We conducted a meta-analysis of the effects of BoNT/A on MDD. The selected studies included the following: (1) clinical depression in participants, (2) sole treatment with BoNT/A glabellar injection, (3) study outcomes of depression scores, and (4) RCT studies only.

The authors conclude that, after considering all the studies mentioned and their own meta-analysis, BoNT/A is a treatment with proven effectiveness, good adherence, a favorable safety profile, and modest costs [62,63,64].

Recently, Moreno-Montoya et al. [65] praised the efforts of researchers to identify techniques for treating depression with fewer side effects. However, I have concluded that this method is not suitable for developing countries because of its high cost.

Feng et al. [66] conducted a study with a 12-week treatment and follow-up period in patients with post-stroke depression. They found that the psychological scores of patients in both the BoNT-A and sertraline groups showed significant reductions compared to baseline levels (*p* < 0.05). The Hamilton Depression Rating Scale (HAM-D) score for the BoNT-A group decreased from 11.63 ± 3.26 (baseline) to 6.69 ± 3.50 (after 12 weeks of treatment), while that for the Sertraline group decreased from 11.30 ± 3.88 (baseline) to 5.91 ± 3.21 (after 12 weeks of treatment). These findings indicate that the effectiveness of BoNT-A in treating depression is comparable to that of sertraline, with no statistically significant differences observed between the two groups.

In this study, performed only on women, we administered 100 U BoNT/A to 20 sites in the frontal, bilateral lateral canthus, and temporal regions. We targeted negative-expression muscles, such as the corrugator and procerus. In addition to these specific muscles, we included the bilateral lateral canthus and bilateral temporal areas to target paralyzed muscles that may block the afferent function of the trigeminal nerve. This blocking effect may reduce the coupling between the brainstem and the left amygdala. However, further research is required to explore lower, safer, and more effective doses for treating patients. The pathogenesis of post-stroke depression is currently the subject of several theories, including neuroanatomy, neurotransmitters, neuroendocrine functions, inflammatory reactions, and psychological factors. Emerging research indicates that the pathogenesis of PSD may involve a disruption of the monoaminergic system, neuroinflammation, and other molecular mechanisms, including a reduced expression of brain-derived neurotrophic factor (BDNF) [66].

Demchenko et al. [67] conducted a systematic review of published studies and ongoing publicly registered clinical trials investigating the efficacy of BONT/A therapy for psychiatric disorders. The authors identified 17 relevant published studies and four unpublished trials conducted over the last 18 years. This demonstrates the significant interest of the scientific community in investigating the therapeutic potential of BoNT/A for the management of psychiatric disorders, including depression, bipolar, social anxiety, and limited personality disorders. Fifteen of the seventeen published studies demonstrated symptom reduction as measured by clinical outcome rating scales specific to the psychiatric indications investigated. One included a study focusing on BONT/A for MDD, which revealed that the concurrent use of other psychotropic medications did not significantly affect the efficacy of BONT/A therapy [68].

Furthermore, two of the included studies focusing on BONT-A for social anxiety and bipolar disorders reported that participants discontinued the use of psychotropic medications because of treatment ineffectiveness or adverse events [69]. Overall, these trends suggest that BONT-A therapy may be the proper therapeutic option for many psychiatric pathologies.

In the present study, Chen et al. [70] investigated the mechanisms by which BoNT/A alleviates neuropathic pain. They demonstrated a significant elevation in SPP1 within microglial cells by establishing models of lipopolysaccharide-induced microglial pyroptosis and spared nerve injury. Furthermore, SPP1 suppression inhibits microglial pyroptosis and alleviates pain in nerve-injured rats. Microglial pyroptosis is a novel form of inflammasome-mediated cell death associated with immune and inflammatory diseases of the central nervous system, including depression, radiation-induced brain injury, PN, and spinal cord injury [70].

NLRP3 inflammasomes play a crucial role in microglial activation. They are predominantly expressed in microglial cells and have been widely studied in the context of neuropathic pain [71].

Shu et al. [72] conducted a prospective randomized study to evaluate the efficacy of BoNT/A in the management of mild-to-moderate depression. A total of 140 females diagnosed with mild-to-moderate depression were enrolled in this study and were randomly assigned to one of two treatment groups, each containing 70 patients who received BoNT/A injections of 100 or 50 units.

The Hamilton Depression (HAM-D), Self-Rating Depression (SDS), Hamilton Anxiety (HAMA), and Self-Rating Anxiety (SAS) scales were used.

The scores reduced considerably after 12 weeks, and the authors found no significant differences between the two groups, suggesting that 50 units were sufficient.

The key findings are summarized in Table 1.

**Figure 3:**
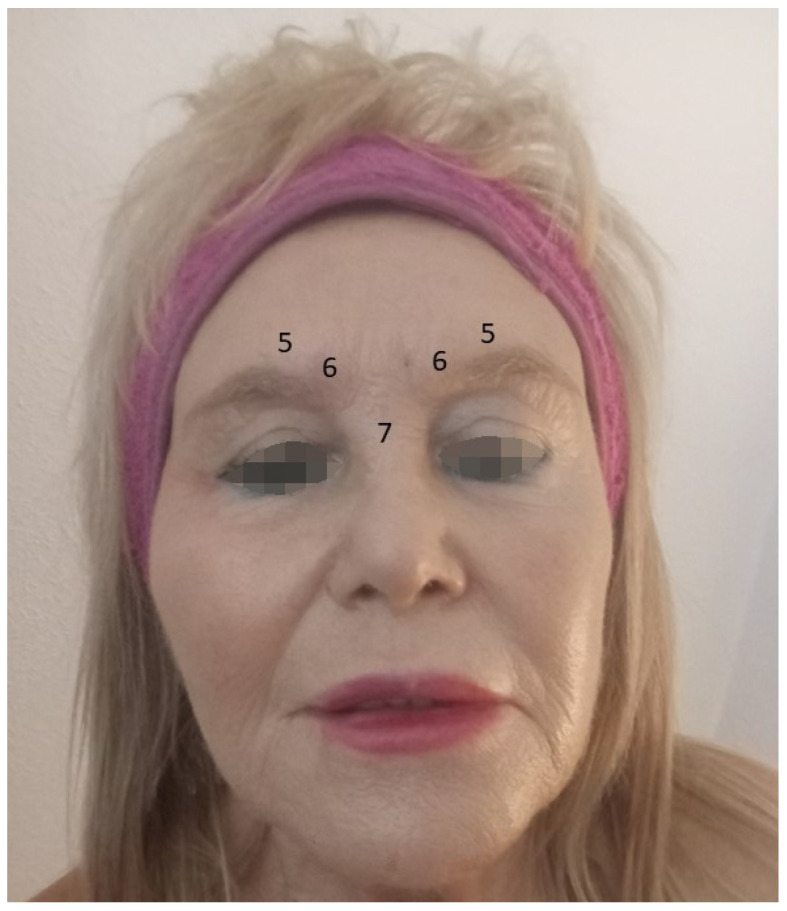
Botulinum toxin A injection schema. The numbers refer to the injected BoNT/A units in women. The numbers are units on injection sites in women.

**Figure 4:**
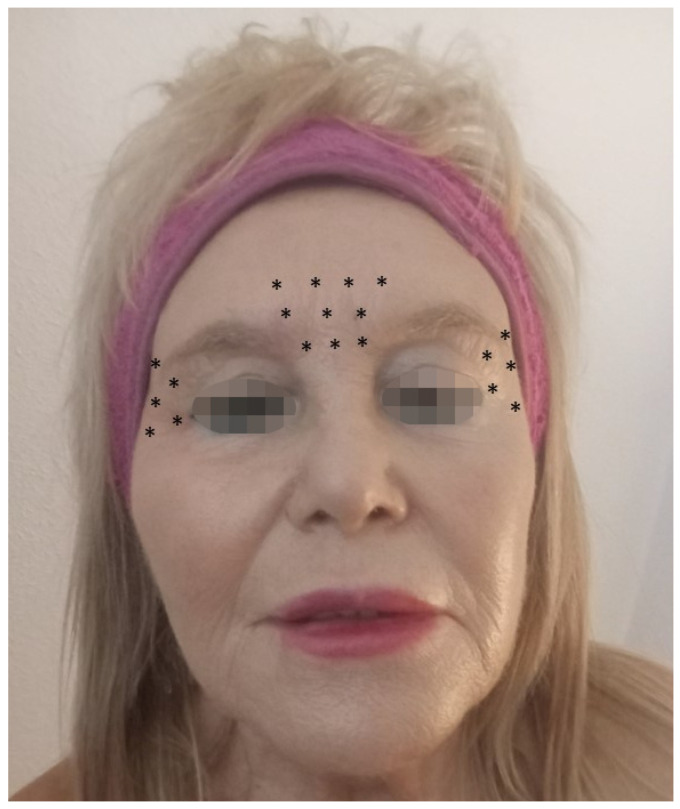
A schematic diagram of BoNT/A injection sites. The occipital frontalis, corrugator, and depressor muscles each received 10 injections, and the bilateral lateral canthus and temporal regions each received 5 injections. A total of 20 sites and 5 units or 2.5 units per site were applied (total 50 or 100 units). The * marks the injection sites in women.

Most authors recommend administering 29 units of OnNT/A distributed at five injection points for the treatment of depression: 7 U procerus muscle, 6 U corrugator muscle, medially bilaterally, and 5 U corrugator muscle, laterally and bilaterally, for women. In men, increasing the dose by two or more units at each injection point is advisable (Figure 3). However, Feng et al. [66] and Shu et al. [72] recommended using 100 units (Figure 4).

Finally, in an experiment carried out by Ceolato et al. [73], it is advisable to add 2–3 U in the depressor anguli oris muscle bilaterally; 3–5 U in the mentalis muscle bilaterally; however, the result was less effective than the application in the glabellar area.

## 4. Conclusions

There is a positive feedback loop between facial expression muscles and the brain. When certain muscles involved in expressing sadness are contracted, the brain interprets this contraction as true sadness and enhances this feeling.

BoNT/A, when applied to the corrugator and procerus muscles, exerts an antidepressant effect by preventing contractions of these muscles. BoNT/A has been shown to have a good safety profile and is effective in treating depression; therefore, it would be interesting to communicate this information to health professionals. In addition, as it is a localized treatment, it avoids the systemic adverse effects associated with antidepressant drugs.

It is essential to continue the research that has already begun in this field. With an increase in studies, it will be possible to assess the positive effects of BoNT-A in both the short- and long-term, as well as outcomes of studies from larger sample sizes, economic profitability, optimal treatment frequency, and its synergistic effect with other therapies or treatments. This will ensure that patients receive the best possible care and treatment grounded on the latest scientific evidence.

Finally, BoNT/A treatment for depression represents a new paradigm in psychodermatology.

## Figures and Tables

**Table 1:** Summary of the reviews carried out, including measurement scales and results.

Author	Rating Scale	Results
Magid et al., 2015 [17]	HAM-D, MADRS	Overall score reduction by 45.7% versus 14.6% in the placebo group.
Parsaik et al., 2016 [52]	HAM-D MADRS, BDI	Depressive symptoms were reduced after treatment with BoNT/A.
Coles et al., 2019 [46]	Combined results for all weeks, measures and types of comparisons	At 6 weeks after the intervention, the BoNT/A groups were significantly less depressed than the placebo groups.
Li et al., 2019 [35]	HAM-D	There was a significant improvement in depressive symptoms of the BONT/A group compared to the placebo group throughout the 12-week follow-up period.
Qian et al., 2020 [47]	HAM-D, BDI	Significant improvement in depressive symptoms after treatment with BoNT/A compared to placebo.
Arnone et al., 2021 [48]	HAM-D, MADRS	Significant reduction in depressive symptoms after treatment with BoNT/A compared to placebo.
Schulze et al., 2021 [62]	HAM-D, MADRS	When comparing time and treatment in a single model, depressive symptoms were reduced more after treatment with BoNT/A compared to placebo (d = 0.98).
Affatato et al., 2021 [53]	DDI BDI-II PHQ-9	OnNT/A leads to a significant improvement of both migraine and depressive symptoms in comorbid patients.
Chen et al., 2021 [70]	----------------------	BoNT/A alleviated neuropathic pain and depression in rats.
Dressler et al., 2022 [61]	------------	Therapy with BoNT/A was applied with fixed interinjection intervals of at least 12 weeks with a significant improvement in both the migraine and depressive symptoms.
Crowley et al., 2022 [55]	HAM-D BDI MADRS	The OnNT/A treatments in all nine studies were associated with a 1.61 SD reduction in depression score. These findings indicate that glabellar OnNT/A injections seem to be an effective treatment of major depression disorders.
Wollmer et al., 2022 [32]	HAM-D17 HAM-D21 MADRS BDI	Six weeks after the baseline, the 29/39 U OnNT/A group showed significantly greater improvement and response rates compared to the saline placebo group.
Finzi et al., 2023 [47]	HAM-D21 HAM-D17 BDI	After 16 weeks, the score was reduced by 47% in those treated with BoNT/A and by 9% in the placebo group.
Kels et al., 2023 [49]	PHQ-9 HAM-D MADRS BDI QIDS SR-16	After 16 weeks, the score was reduced by at least 50%. Remission is identified by sub-threshold scores.
Moreno-Montoya et al., 2023 [65]	----------------------	They briefly review the treatment, indicating that it is good but inappropriate for developing countries.
Feng et al., 2024 [66]	HAM-D SDS HAMA SAS	They used the 12-week time point as the evaluation endpoint. The score was considerably reduced with 100 units of BoNT/A.
Demchenko et al., 2024 [67]	BDI HAM-D MADRS HAMA SDS SAS	After 12 weeks, the score was considerably reduced in most of the published articles. Men often require higher doses than women.
Shu et al., 2024 [72]	HAM-D SDS HAMA SAS	After 12 weeks, the score was considerably reduced with both doses, 50 or 100 units of BoNT/A.

BoNT/A: botulin toxin A; BDI: Beck Depression Inventory; BDI-II: Beck Depression Inventory II; HAM-D: Hamilton Depression Rating Scale; HAM-D17: Hamilton Depression Rating Scale 17; HAM-D21: Hamilton Depression Rating Scale 21; HAMA: Hamilton Anxiety Scale; MADRS: Montgomery–Asberg Depression Rating Scale; PHQ-9: Patient Health Questionnaire 9; QIDS SR-16: Quick Inventory of Depressive Symptomatology Self-Report; SAS: Self-Rating Anxiety Scale; SDS: Self-Rating Depression Scale.
